# Treatment of Diarrhoea in Rural African Communities: An Overview of Measures to Maximise the Medicinal Potentials of Indigenous Plants

**DOI:** 10.3390/ijerph9113911

**Published:** 2012-10-26

**Authors:** Collise Njume, Nomalungelo I. Goduka

**Affiliations:** 1 Department of Medical Microbiology, Walter Sisulu University, Mthatha 5117, South Africa; 2 Centre for Rural Development, Enkululekweni, Walter Sisulu University, Mthatha 5117, South Africa; Email: ngoduka@wsu.ac.za

**Keywords:** diarrhea, gastrointestinal transit, indigenous medicinal plants, health-promotion efforts, rural Africa

## Abstract

Diarrhoea is a major cause of morbidity and mortality in rural communities in Africa, particularly in children under the age of five. This calls for the development of cost effective alternative strategies such as the use of herbal drugs in the treatment of diarrhoea in these communities. Expenses associated with the use of orthodox medicines have generated renewed interest and reliance on indigenous medicinal plants in the treatment and management of diarrhoeal infections in rural communities. The properties of many phenolic constituents of medicinal plants such as their ability to inhibit enteropooling and delay gastrointestinal transit are very useful in the control of diarrhoea, but problems such as scarcity of valuable medicinal plants, lack of standardization of methods of preparation, poor storage conditions and incertitude in some traditional health practitioners are issues that affect the efficacy and the practice of traditional medicine in rural African communities. This review appraises the current strategies used in the treatment of diarrhoea according to the Western orthodox and indigenous African health-care systems and points out major areas that could be targeted by health-promotion efforts as a means to improve management and alleviate suffering associated with diarrhoea in rural areas of the developing world. Community education and research with indigenous knowledge holders on ways to maximise the medicinal potentials in indigenous plants could improve diarrhoea management in African rural communities.

## 1. Introduction

Diarrhoea is the frequent passage of unformed, loose or watery stools, usually three or more times in 24 hours [1,2]. It is the most common clinical manifestation of gastrointestinal disease and can be caused by both infectious and non-infectious agents [1]. The onset of the disease may be abrupt and self-limiting in immune-competent individuals, but chronic diarrhoea may be persistent even with therapy, especially in people with an underlying debilitating clinical condition such as HIV/AIDS and diabetes mellitus or individuals with an aging immunity [3]. 

Worldwide, an estimated nine million children, most of them younger than 5 years of age die annually as a result of diarrhoea [4,5]. The majority of deaths occur in rural African communities where health care facilities are inadequate and the majority of the people lack access to clean and safe water, a major vehicle for transmission of diarrhoeal diseases [6,7]. It is estimated that diarrhoea kills more young children around the World than malaria, acquired immunodeficiency syndrome (AIDS) and tuberculosis combined [7]. In some rural parts of the developing world, the mother’s knowledge on the predisposing factors of diarrhoea are poor and at times the frequent occurrence of childhood diarrhoea is wrongly perceived as a developmental stage of the child and at times virtually results in mortality [7]. 

Antimicrobial chemotherapy can shorten both the bacterial excretion and duration of illness [2]. However, this will depend on where the patient/guardian chooses to seek health care and also the individual’s perception of the disease. There is a relationship between the mothers’ perceptions of diarrhoea and treatments sought [8]. For example, in some parts of Africa, severe diarrhoea is believed to be caused by an external agent such as a spirit or jealous person and therefore treated with traditional therapies related to these perceived causes [9]. In Tanzania, severe cases of childhood diarrhoea are often attributed to transgressions of sexual taboos by the parents, especially mothers, and therefore treated by some form of cleansing [10]. Such cases are culture-related and may fail to respond to modern forms of treatment.

Many researchers have associated the high incidence of diarrhoea in Sub Saharan Africa to poverty [11], a fact that is supported by its high morbidity and mortality in rural low-income communities. Living in socio-economic-deprived neighbourhoods is reported to be associated with less likelihood of seeking less medical care [11]. Apart from cost of treatment, diarrhoeal pathogens are becoming distressingly resistant to antimicrobial agents and some of the major treatments of diarrhoea (oral rehydration solutions, ORS) may not reduce the volume of stool or duration of illness [12]. As a means of ensuring effective treatment therefore, clinicians have resorted to the use of combination therapies in the management of diarrhoeal diseases leading to increase pill burden and longer duration of therapy. Considering that these therapies may still be ineffective and adverse effects may occur, and given the fact that hospitals may not be readily accessible in rural African communities, dependence on indigenous herbal medicines as remedies for diarrhoea is on the rise in these communities. 

There are two major health care systems used in the treatment of diarrhoea in the developing world, orthodox and indigenous systems [13]. While the orthodox system is well structured and highly developed, the indigenous systems are poorly organised and virtually unregulated [14]. The latter make use of informal methods of treatment, some of which are considered remote and inefficient. Both systems often exist side by side, but seldom cooperate, probably due to the fact that certain aspects of indigenous medicine often rely on mysticism and intangible forces such as witchcraft, and some other aspects are based on spiritual and moral principles [15]. While these may be valid psychologically, they are difficult to rationalize scientifically [15]. In any case, more than 80% of people in rural African communities still rely on indigenous medicine as a primary source of health care [14,16]. This is partly due to the fact that the majority of the people are not able to meet the high cost associated with the western health care system and also the benevolent attachment to their culture and tradition [17]. The World Health Organisation (WHO) has therefore encouraged interaction between western-based and indigenous-based medicines with a view to exploit and identify compounds that could provide safe and effective remedies for ailments of both microbial and non-microbial origins [18]. 

The ratio of indigenous healers to the population in Sub Saharan Africa is approximately 1:500, while medical doctors have a 1:40,000 ratio to the rest of the population [17]. This is an indication that an overflow of people to the indigenous health system is almost always inevitable. Considering that indigenous medicine is one of the most commonly sought remedies for treating diarrhoea in many rural areas of the developing world, it becomes imperative to pursue measures to maximise the medicinal potential of indigenous plants. Such measures would help alleviate undue suffering associated with the disease and reduce the number of lives lost. This review examines useful medicinal plants employed in the control of diarrhoea in rural African communities and points out major areas that could be targeted by health-promotion efforts as a means to improve management and alleviate the suffering associated with this disease. 

## 2. Aetiology of Diarrhoea

Diarrhoea is a common disease accounting for up to 75% of childhood illnesses in Africa with a severity that seems to depend to some extent on aetiology and age [19]. The causes of diarrhoea are wide and varied, the majority of them are related to poor sanitary conditions and low socio-economic status [2,11]. Infectious diarrhoea, the most common form of diarrhoea worldwide may be caused by viruses, bacteria or protozoa [1,20]. Examples of viral causes of diarrhoea include Rotavirus, Enteric Adenovirus, Norovirus, Enteroviruses, Caliciviruses and Astroviruses [21]. Worldwide, Rotavirus infection is responsible for the most severe forms of diarrhoea, especially in children and may account for up to 40% of cases in the developed countries and 25% in the developing world [11,21]. Infections with bacteria such as enterotoxigenic *Escherichia coli* (ETEC) and *Campylobacter jejuni* account for 25% and 18% of diarrhoeal cases in the developing world, respectively [11]. Diarrhoeagenic *E*. *coli* (DEC) which include enteroinvasive *E*. *coli* (EIEC), enteroaggregative *E*. *coli* (EAEC), enterohaemorrhagic *E*. *coli* (EHEC), enteropathogenic *E*. *coli* (EPEC) and ETEC are among the major bacterial causes of diarrhoea in the world [22]. Other bacterial causal agents include *Vibrio cholerae*, non-typhoidal *Salmonella*, *Shigella* species and *Salmonella typhi* [1,21]. Protozoans such as *Cryptosporidium parvum*, *Giardia lamblia* and *Entamoeba histolytica* have also been incriminated as serious causes of diarrhoea in Africa and other parts of the developing world [23,24]. A recent study in West Africa reports *Giardia lamblia* as the major parasitic cause of childhood diarrhoea in the Ashanti Akim North municipality of Ghana, with a prevalence of 89.5% [24]. Other researchers have also reported the role of other organisms such as *Entamoeba histolytica* and *Cryptosporidium parvum* as major causes of diarrhoea in other African communities, most of them characterised by over-crowding and poor environmental sanitation [25,26]. Non-infectious diarrhoea may be caused by toxins, poisons, drugs and sometimes food allergens. Prolonged use of antibiotics resulting in the disruption of gut microflora may cause diarrhoea and at times pseudomembranous colitis resulting from *Clostridium difficile* infection. However, the occurrence of diarrhoea may at times also be indicative of clinical conditions that are located out of the gastrointestinal tract [2]. Reactions to some magnesium-containing drugs (e.g., antacids) may cause mal-digestion or mal-absorption sometimes stimulating the expulsion of gut contents. Drug-related diarrhoea is the most common form of diarrhoea in the elderly probably due to the fact that major organs of drug clearance such as the kidneys and liver are affected by age [3].

Irrespective of aetiology, diarrhoea most of the time will occur when there is an imbalance between absorption and secretion, when the absorptive capacity of the intestine is exceeded and net secretion is greater than absorption [3]. Worthy of note is the fact that even minimal changes in normal intestinal fluid and electrolyte balance may result in diarrhoea [27]. Such changes may be caused by infectious agents, toxins, and other noxious agents present in the gut causing disruption of normal fluid secretion and stimulating the gut to expel its contents. This response is protective for acute irritations of the gut but becomes an issue when chronically present and no longer serving a physiological role [28]. Due to failures in the regulation of ionic balance, differences in fluid absorption and secretion may result in very large changes in stool consistency and volume. Excessive loss of fluids, electrolytes and nutrients due to diarrhoea may result in dehydration, acidosis, malnutrition and haemolytic uremic syndrome [20,21]. In the elderly, limited physiological reserves and co-existing diseases have been reported to increase the frequency and severity of diarrhoeal complications such as dehydration and electrolyte loss, known to be responsible for most hospitalizations and fatalities in nursing homes and acute care facilities [3].

The ingestion of poorly absorbable low molecular weight aqueous solutes may create an osmotic pressure which pulls water and ions into the intestinal lumen and instigates frequent stooling leading to osmotic diarrhoea [20,29]. This kind of diarrhoea can also be caused when individuals with congenital lactase deficiency consume lactose-rich diets [20]. If the intestinal epithelium’s barrier function is compromised by loss of epithelial cells or disruption of tight junctions, hydrostatic pressure in blood vessels and lymphatics will cause water and electrolytes, mucus, protein, and sometimes even red and white cells to accumulate in the lumen leading to watery stools [30,31]. This kind of diarrhoea is called exudative diarrhoea and is common in bacterial infection, especially *Shigella* [31]. Other organisms associated with exudative diarrhoea include *Salmonella*, *Yersinia*, *Campylobacter*, *Aeromonas*, Entero-invasive *E*. *coli* and *Rotavirus* infection [27]. These organisms invade the epithelium and multiply, damaging the surface epithelium and causing inflammation [31]. Diarrhoea is due both to the epithelial damage (exudation and decreased absorptive capacity) and to the action of inflammatory mediators. Common forms of diarrhoea as reported by different investigators are represented on Table 1.

**Table 1:** Common forms of diarrhoea classified according to causative agent, pathogenic mechanism and clinical features.

Aetiology	Main site of action	Primary mechanism	Clinical features	Reference(s)
ETEC	Small intestine	Heat stable and heat labile toxins produced by the organism induce secretory diarrhoea	Watery stools associated with fever, abdominal cramps and vomiting	[22,27]
EPEC	Proximal small intestine	Attachment/effacement of enterocytes, alteration of intracellular calcium and cytoskeleton	Self-limiting watery diarrhoea occasionally accompanied with fever and vomiting.	[22,27]
EIEC	Distal ileum and colon	Tissue invasion and mucosal destruction	Watery occasionally bloody diarrhoea	[27]
EHEC	Colon	Elaboration of potent shiga-like cytotoxins l and ll	Bloody diarrhoea in 90% of cases and haemolytic uremic syndrome in 10%.	[22,27]
*Vibrio* *cholerae* enterotoxin	Endocrine cells on the villus surface of the intestinal epithelium	Enterotoxins cause an increase in cAMP or cGMP inducing cAMP-mediated alterations of ion transport.	Voluminous watery diarrhoea without abdominal cramps or fever; nausea and vomiting.	[31]
*Shigella*	M-cells of the colonic and rectal epithelium	Bacteria invade the intestinal epithelium damaging it and causing inflammation. Diarrhoea is due to epithelial damage and inflammatory mediators.	Abdominal cramps and pain with initial high volume watery stool that eventually reduces in volume, becomes stained with mucus and blood and associated with urgency and painful defecation.	[32,33]
*Salmonella*	Peyers patches of the small intestine	Bacteria invade the intestinal epithelium damaging it and causing inflammation. Diarrhoea is due to epithelial damage and inflammatory mediators.	Loose stools to profuse watery diarrhoea, nausea, vomiting and sometimes persistent headache, especially in *S*. *typhi* infection.	[33]
*Yersinia enterocolitica*	Intestinal epithelium of the terminal portion of the ileum	Bacteria invade the intestinal epithelium damaging it but inhibit host inflammatory responses	Abdominal pain and diarrhoea occasionally accompanied by fever, nausea, vomiting and malaise.	[33,34]
Norovirus	Sub-mucosa of proximal small intestines	Continuous viral replication in the sub-mucosa of the proximal small intestines is believed to interfere with normal intestinal function	Stomach pain, fever, nausea, vomiting, mild self-limiting and non bloody diarrhoea	[37,38]
Astroviruses	Epithelial cells of the proximal small intestines	Viral infection increases intestinal barrier permeability and causes sodium mal-absorption creating an osmotic pressure which pulls water and ions into the intestinal lumen.	Moderate to severe diarrhoea characterised by abdominal pain and vomiting.	[29,30]
Enteric adenoviruses	Intestinal epithelium, peyers patches in the ileum	Viral infection of the intestinal epithelium damages endothelial cells and interferes with smooth functioning of the intestines	Watery diarrhoea accompanied by vomiting, low grade fever and mild dehydration.	[39,40]
Cytomegalovirus	Entire gastrointestinal tract but frequently involves the oesophagus and colon	Viral infection causes intestinal inflammation, erosion and ulceration with inclusions in the stromal and endothelial cells. Causes distal oesophageal ulceration.	Acute watery diarrhoea, stained with blood and may be persistent	[5,41]
*Cryptosporidium parvum*	Surface epithelial cells lining the distal jejunum and ileum.	Protozoan invades minimally the intestinal mucosa causing self-limiting diarrhoea in immune competent individuals.	Mild to severe watery diarrhoea	[42]
*Giardia lamblia*	Small intestine	Colonisation of the intestine is an important step for diarrhoea. Initially, there is excystation followed by attachment to the intestinal epithelium and multiplication, then encystment. This process disrupts and distorts the microvilli of the intestine.	Asymptomatic, Stools are loose or semi-formed, mild abdominal discomfort	[24,43]
*Entamoeba histolytica*	Small intestines	Ingested cysts rupture in the small intestine releasing trophozoites which invade the mucin layer of the intestinal mucosa. Protozoan has an ability to kill and phagocytise host cells.	Lumpy mucoid stools with blood stains, diarrhoea, cramping, abdominal pain, flatulence, tenesmus rectal, headache and vomiting	[44,45]
*Balantidium coli*	Caecum and colon	Trophozoites produce proteolytic enzymes that digest the mucus coating of the colon facilitating tissue invasion, abscess formation, ulceration and perforation of the intestine.	Acute explosive watery diarrhoea, stools may be stained with blood. Cramping, halitosis, abdominal pain. Tenesmus, weight loss and intestinal perforations are seen in severe cases.	[5,46]

ETEC, enterotoxigenic *E*. *coli*, EPEC, enteropathogenic *E*. *coli*, EIEC, enteroinvasive *E*. *coli*, EHEC, enterohaemorrhagic *E*. *coli*.

## 3. Diagnosis of Diarrhoea

Frequent passage of loose or watery stools 3–4 times a day is indicative of diarrhoea. Laboratory diagnosis is usually achieved by examination and culture of stool specimens [47]. Wet mount preparations of stool samples observed under 40× magnifications are used to view parasites as well as their ova [24]. Gram-stained slides may also reveal greater details of the bacteria flora and presence of yeast. Stool samples are inoculated into bacteriological media, incubated and cultures identified using standard microbiological and biochemical techniques [47]. Identification of viral agents is usually achieved through tissue culture studies. Different variations of nucleic acid-based methods including the polymerase chain reaction (PCR) have been widely employed in the laboratory identification of diarrhoeic agents due to their rapidity, sensitivity and specificity [47]. However, their application is also limited by the high cost of equipment, lack of expertise and false positive results that may arise from stool contaminants or poorly processed equipment [48]. 

## 4. Treatment Strategies

The objective of any anti-diarrhoeal treatment is to replace or minimise fluid and electrolyte loss, reduce stool frequency and any other symptoms such as abdominal pain, reduce faecal losses and ultimately reduce duration and severity of illness [20]. Therefore, the administration of oral rehydration solutions (ORS) to replace fluid and electrolyte loss in diarrhoeic patients is sine qua non to effective treatment [20]. Different formulations of these solutions exist but the basic ingredients are water, electrolytes (e.g., sodium) and glucose. Their mechanism of action lies in the fact that sodium/glucose co-transport proteins on the brush boarder cells of the intestinal lumen pull sodium and glucose from the gut into the cells [49]. As the cellular osmotic pressure increases, water is reabsorbed out of the gut into the body. This action reverses electrolyte imbalances and re-hydrates the patient. Rice starch and other concentrated carbohydrates are also being used in the newer formulations of ORS with the advantage that an increased amount of cellular substrate will also drive active sodium absorption bringing about relief of symptoms [50]. Combined administration of ORS and zinc has also been reported to alleviate diarrhoeal symptoms and expedite recovery of many patients in different parts of the world [51]. This treatment is being encouraged because it may be a way to avoid unnecessary use of antibiotics, especially in children. Furthermore, a 10–14 day course of zinc during and after diarrhoea has been reported to decrease the recurrence of the disease in the next 2–3 months [9,51]. Despite the relief obtained with ORS, lack of parental knowledge concerning their application is among the major factors that limit their usage in rural and semi-urban areas of the developing world. It is also difficult to administer the therapy successfully to patients whose purging episodes are accompanied by vomiting [20]. In this case, intravenous fluid replacement by a professional medical staff may be required. Such staffs are difficult to find in African rural communities. 

### 4.1. Anti-Motility and Anti-Secretory Agents

Anti-motility agents (e.g., loperamide and diphenoxylate-atropine combinations) act by increasing intestinal transit time and enhancing the potential for re-absorption of fluids and electrolytes [20]. However, these groups of drugs are usually not recommended for children and young infants due to the potential of central nervous system side-effects. The anti-secretory properties of bismuth salicylate have been shown to be effective in reducing the number of unformed stools by approximately 50% in patients with travellers’ diarrhoea [20,52]. Apart from its anti-secretory properties, bismuth salicylate also has antibacterial and anti-inflammatory properties which make it a good candidate for the treatment of diarrhoea. However, this drug is not a very popular choice because of its high pill burden, delayed onset of action and the presence of unpleasant side-effects such as tinnitus and black tongue [52,53].

### 4.2. Antimicrobial Therapy

Antimicrobial therapy shortens the duration of the illness, prevents development of complications and reduces the severity of associated symptoms such as fever and abdominal pain [54]. It also decreases secondary cases by halting person-to-person spread of diarrhoeic pathogens. However, the use of antibiotics in the treatment of diarrhoea is being approached with caution due to potential problems of drug-resistance, side-effects and cost of treatment [55]. There is also the fear that antibiotic therapy may worsen the clinical state of the patients because of their effect on gut micro-flora. In most cases, antibiotic treatment is only recommended in the treatment of acute bloody diarrhoea in children [21,54]. When prescribing antibiotics for the treating of diarrhea, clinicians have not only to be aware of the most likely pathogens, but also of their antimicrobial susceptibility patterns and safety profiles [54]. 

### 4.3. Treatment with Indigenous Herbal Medicines

Medicinal remedies prepared from indigenous plants are almost always the only readily accessible and affordable therapies for the control of diarrhoea in many rural communities in the developing world [56]. In these communities, extracts, decoctions/concoctions or ashes of various plant parts (roots, rhizomes, tubers, aerial parts, stem barks and leaves) are used as remedies for diarrhoea and other illnesses [48]. The literature is rich with information on the anti-diarrhoeal activities of most of these plants and some have been scientifically validated, with isolated active components [1,17,57,58]. The anti-diarrhoeal activity of many of the plants have been found to be due to the presence of tannins, alkaloids, saponins, flavonoids, steroids and/or terpenoids [58,59]. However, only few of these compounds have eventually found themselves on pharmaceutical shelves as anti-diarrhoeal agents after several years of testing and clinical evaluation. These findings lend pharmacological credence to the anecdotal, ethno medicinal use of medicinal plants as remedies for diarrhoea and indicate the need for more research in this area. Of the 350,000 plant species found in the world, only about 20–30% have been investigated thoroughly and only between 5–10% are currently known to be used in traditional medicine [60]. At least two out of every 10 medicines prescribed in hospitals are derived from plants, most of them discovered through the use of indigenous medicinal plants [61]. It is also estimated that at least seven out of every 10 medicines used in the treatment of cancer have been derived from medicinal plants [62,63]. About 10% of the world’s terrestrial plants are found in South Africa, many of which are used as herbal medicines and still largely unexplored [56,64]. There is therefore an urgent need for rigorous research in South Africa’s flora as it offers the potential for the discovery of new drugs, thus making significant contributions in the country’s health care system.

**Table 2:** Indigenous medicinal plants employed in the treatment of diarrhoea in rural and semi-urban areas of the developing world.

Scientific name	Family	Part(s) used	Preparation	Phytochemical Ingredients and Mechanisms of action	Reference (s)
*Psidium guajava* Linn	Myrtaceae	Leaves	Infusion/Decoction	Antimicrobial, prevents attachment and colonisation of bacteria to the intestinal epithelium. Interferes with bacterial metabolism. Antispasmodic activity, inhibition of increased watery secretion and inhibition of acetylcholine release. Inhibition of intestinal transit through the inhibition of PGE2-induced enteropooling	[59,65,66,67,68]
*Conyza dioscoridis* (L.) Desf.	Compositae	Leaves	Decoction	The phyto-component-rich ethanol extract has been shown to induce a dose-dependent relaxation of duodenal muscles possibly through calcium-channel and ganglionic blocking effects.	[69]
*Tithonia diversifolia*	Asteraceae	Leaves	decoction	Phytochemicals have antispasmodic activity.	[70,71]
*Alchornea cordifolia* Shum and Thon.	Euphorbiaceae	Leaves	Decoction	Phytochemical components such as tannins and flavonoids are thought to delay intestinal transit, increase colonic water and electrolyte reabsorption by modifying their transport across the colonic mucosa. Ethyl acetate extracts also have antimicrobial properties against diarrheagenic organisms such as *E*. *coli*, *Candida albicans*, *Staphylococcus aureus* and *Pseudomonas aeruginosa*.	[72,73]
*Heinsia pulchella* K. Shum		Root bark	Decoction	Plant crude extracts contain various phytochemical components with antimicrobial activity against diarrhoea-causing organisms such as *Entamoeba histolytica*.	[74,75]
*Catharanthus roseus* Linn	Apocyanaceae	Leaves	Decoction	Plant is rich in tannins, alkaloids, flavonoids, saponins and triterpenes some of which have anti-motility effects by their inhibition of gastrointestinal transit.	
*Alhagi maurorum* Medic.	Leguminosae	Aerial parts	Decoction	Phytochemical constituents such as flavonoids, tannins and unsaturated sterols/terpenes may cause a relaxing effect on the intestinal tissue possibly through calcium channel blocking effect.	[69]
*Mentha microphylla* C. Koch.	Labiatae	Aerial parts	Decoction	Phytochemical constituents such as flavonoids, tannins and unsaturated sterols/terpenes may cause a relaxing effect on the intestinal tissue possibly through calcium channel blocking effect.	[69]
*Zygophyllum album* L.f. Täckh.	Zygophyllaceae	Aerial parts	Decoction	Phyto-constituents in the plant extracts cause relaxation of duodenal muscles possibly through calcium-channel and ganglionic blocking effects.	[69]
*Cylicodiscus gabunensis*	Mimosaceae	Stem bark	Decoction	The plant may contain protein tannates that may make the intestinal mucosa more resistant, thus reducing intestinal secretion, increase reabsorption of water and decrease motility.	[76,77]
*Euphorbia hirta*	Euphorbiaceae	Leaves	Macerate	Plant contains the flavonoid glycoside quercitrin known to exhibit anti-motility effects, delay intestinal transit and increase colonic fluid absorption when combined with secretagogue compounds such as PGE2 and sodium picosulphate.	[78,79]
*Hippocratea africana*	Hippocrateaceae	Roots	Decoction	Plant contains phytochemical components that inhibit intestinal transit and fluid accumulation in the small intestines.	[80]
*Securinega virosa*	Euphorbiaceae	Roots, leaves and stem bark	Decoction	Phytochemical components such as anthraquinone glycosides, alkaloids, tannins, saponins and alkaloids present in different parts of the plant cause contraction of jejunal tissue and inhibition of prostaglandin biosynthesis.	[81]
*Ziziphus mauritiana* Lam	Rhamnaceae	Root	Decoction	Phytochemicals in the plant may cause inhibition of acetylcholine-induced contraction of the ileum, decrease gastrointestinal transit and inhibit fluid accumulation in the intestines.	[82]
*Mormodica charantia*	Curcubitaceae	Leaves	Decoction	The plant is believed to contain phytocomponents with morphine-like action against diarrhoea. The major mechanisms being their ability to delay gastrointestinal propulsion, reduce number of stools and inhibit intestinal fluid accumulation.	[83]
*Stereospermum kunthianum *(Cham, Sandrine Petit)	Bignoniaceae	Stem bark	Decoction	Plant contains phytochemicals that are believed to delay intestinal transit.	[84]
*Bridelia micrantha*	Phyllanthaceae	Stem bark	Decoction	Phytochemicals in the plant cause inhibition of intestinal transit through the inhibition of PGE2-induced enteropooling.	[66]
*Eleutherina bulbosa *(Mill.)	Iridaceae	Bulb	Decoction	Phytochemicals in the plant cause inhibition of intestinal transit through the inhibition of PGE2-induced enteropooling.	[66]
*Dissotis rotundifolia* Triania	Melastomaceae	Leaves	Decoction	Plant is rich in alkaloids, tannins, cardiac glycosides reported to be antimicrobial against diarrhoea-causing organisms. Some of these compounds also inhibit intestinal motility.	[85]
*Trilepisium madagascariense* DC, Leeuwenberg	Moraceae	Stem bark	Decoction	The plant is rich in phytochemical compounds. A compound isolated from this plant, isoliquiritigenin is reported to inhibit diarrhoea droppings by 84.81%. The methanol extract reduces enteropooling , inhibit gastrointestinal motility in *Shigella*- and Castor oil-induced diarrhoea in rats by increasing reabsorption of electrolytes and water or by inhibiting induced intestinal accumulation of fluid just as the standard drugs loperamide and diphenoxylate HCl.	[58]
*Carica papaya*	Caricaceae	Seeds	Decoction	Phytochemicals in the seeds have been reported to demonstrate antimicrobial activity against many diarrhoea-causing bacteria such as *Salmonella enteritidis*, *Escherichia coli*, *Shigella flexneri* and opportunistic pathogens.	[70]

It is also important to isolate and study medicinal plant components considering that valuable medicinal plants are often overexploited and this might lead to extinction. There is the fear that the multitude of potentially useful phytochemical components which could be synthesized chemically may be at risk of being lost irretrievably [65]. Represented on Table 2 are some of the medicinal plants commonly employed in the treatment of diarrhoea in rural and semi-urban communities of the developing world. For the most part, only plants used in African indigenous medicine and scientifically validated, most of the time in animal models as having anti-diarrhoeal properties are presented. 

#### 4.3.1. Pharmaceutical Anti-Diarrhoeic Agents Isolated from Medicinal Plants

Before the introduction of Western orthodox medicine, indigenous medicinal plants were and are still the main form of treatment of diarrhoea and other diseases amongst many rural communities in Africa [86]. It has become crystal clear in recent times that most of the plants and herbs used in traditional medicine could constitute a great source of useful therapeutic compounds [87]. Researchers have documented a huge list and databases of therapeutically useful compounds isolated from plants, most of which form an important pillar in the practice of modern allopathic medicine. In this paper, we also examine some of the chemotherapeutic agents that originate from the use of indigenous herbal remedies, the findings of which are summarised in Table 3. For the most part, only drugs used in the treatment of dysentery or diarrhoea-like diseases are represented on this table. 

**Table 3:** Pharmaceutical products derived from indigenous medicinal plants used in the control of dysentery or diarrhoea-like conditions.

Drug/Chemical	Specific Use	Plant
Aesculetin	Anti-dysentery	*Frazinus rhychophylla*
Emetine	Amoebic dysentery	*Cephaelis ipecacuanha*
Agrimophol	Anthelmintic	*Agrimonia supatoria*
Berberine	Bacillary dysentery	*Berberis vulgaris*
Hemsleyadin	Bacillary dysentery	*Hemsleya amabilis*
Neoandrographolide	Dysentery	*Andrographis paniculata*

A careful look at this table indicates that not many anti-diarrhoea drugs are produced from plants. This is surprising considering the wide variety of active plants that are employed for the treatment of diarrhoea, especially in the developing world [5,48,59,66]. It is likely that most of the studies done on medicinal plants have concentrated on the isolation of compounds for the treatment of cancer, cardiovascular diseases and other infections of middle/high-income societies, neglecting diseases of low, overcrowded rural African communities like diarrhoea. Secondly, diarrhoea as earlier indicated may just be a manifestation of an infection located out of the gastrointestinal tract and so most of the drugs targeting such conditions are not included in the table.

## 5. Measures to Maximise the Medicinal Potentials of Indigenous Plants Used in the Treatment of Diarrhoea in Africa

Medicinal plants are a source of hope for African rural dwellers who most of the time cannot meet the ever increasing cost of modern allopathic medicine. The effectiveness of indigenous herbal medicines used in the treatment of diarrhoea is gaining popularity and the hopes of preserving the natural flora is chattered by the day due to the ever increasing demand for the plants employed [88]. In a bid to make a contribution towards the preservation of this very rich and natural source of health, several problems have been identified in this review and measures to enhance the efficacy of the medicines have been proposed. 

### 5.1. Plant Over-Exploitation

Most medicinal plants are harvested from the wild and are often readily available. However, uncontrolled harvesting of large quantities especially for commercial purposes would result in the extinction of many forest plants especially when endemic species with a restricted geographic distribution are included. For example, *Siphonochilus aethiopicus*, *Warburgia salutaris*, *Securidaca longipendunculata* and some *Xysmalobium* species which were readily available in the South African biota are now at the verge of extinction because of over-exploitation [89,90]. In the past, harvesting of medicinal plants was done by trained indigenous practitioners who respected customary conservation practices such as seasonal/social restrictions, taboos, limitation of harvested quantities and special harvesting equipment [91]. However, this is not the case with medicine vendors whose main aim is to make profit with no regards to quantities and methods of harvest. In order to overcome this problem, protection of endangered varieties alongside education of the community should be enforced by the local and national governments. Dealers in medicinal plants should beware that plants harvested in different seasons are very likely to contain different ingredients [92,93]. The plants should therefore be collected in the proper season to ensure best quality of both starting material and the finished product. Plants should not be harvested in areas prone to contamination such as roadside paths, drainages, mine tailings and garbage dumps. Nurseries and seed banks should be developed and these alongside appropriate propagation materials distributed to indigenous health practitioners and rural dwellers for cultivation of endangered species in their home gardens. 

Pharmaceutical companies and research institutions should be encouraged to go into cultivation of useful medicinal plants as they may have better control over the supply of plant material and ensure the application of post-harvest treatments. This will go a long way to reduce the dependence on wild varieties, save populations at risk of extinction and conserve genetic diversity [91]. Rural communities and indigenous health practitioners should be educated on the advantages of cultivating valuable medicinal plants in home gardens which may include accessibility, high yields and good quality products. Growing the plants in a home garden is an indication that seeds and seedlings will be readily available and if grown on a large scale could constitute a source of revenue in the home, thus improving rural livelihoods. However, the feasibility of being successful with a home garden will depend on the ability of the species to thrive under mono culturing conditions. The economic viability will depend on the demand and market prices. Where possible, scientific studies to comparatively evaluate the medicinal potential and chemical composition of both wild and cultivated varieties should be investigated [94]. 

### 5.2. The Excessive Harvest of Certain Plant Parts

The parts harvested and the methods employed may also be destructive to the plants [90]. Although the concentration of phytochemical components may vary with plant parts, studies should be done in this area to encourage the use of parts that would enhance sustainability. A good example of one such study conducted by Eloff [95] on the antibacterial activity of *Sclerocarya birrea* revealed that the same compounds that are responsible for antimicrobial activity in the stem bark can also be found in the leaves of the same plant. The use of leaves over stem barks, roots, tubers or whole plants should therefore be encouraged to promote sustainability of endangered species [86]. Non-destructive practices should be employed during harvest of plants. For example, when collecting roots, the main root should not be cut or dug out. Harvesting bark from the branches rather than trunk could be more sustainable to the plant and even when the harvest is done from the trunk, the tree should not be completely stripped of its back. Different trees can take different amounts of time to regenerate the bark [91]. Methods to ensure fast regeneration of bark should be taught in the community and precautions taken to protect the tree while the bark is removed.

### 5.3. Lack of Standardization of Methods

There is a lack of systemic approach when it comes to traditional medicine practice in African rural communities. Just like modern allopathic medicine, traditional medicine can be practiced under very controlled circumstances, using scientifically validated medicines, paying attention to hygiene, prescribing accurate dosages with the awareness of any potential side effects obtained through toxicity studies. Methods of preparation of indigenous herbal drugs for diarrhoea should be standardized considering the importance of these in obtaining effective herbal medicines with the desired effects. Medicinal properties of the plants are closely related to their phenolic constituents [1]. Each constituent may have a preferred method of treatment which may in tend facilitate maximum activity [96]. For example, boiling in hot water or preparing decoctions may extract a group of anti-inflammatory steroids as the hot water penetrates deep into the plant tissues dislodging many active constituents while alcohol preparation may produce antibacterial steroids which may be more effective in combating infective diarrhoea. A juice of the plant may be recommended instead of a decoction/powder if the active anti-diarrhoeic principles are volatile. The use of herbal teas as remedies for diarrhoea instead of cold water extracts may be preferred because they may serve as a means of providing a safe supply of liquids given that the water is boiled and the herbs themselves are made of several phytochemical components, some of which have been proven to have antispasmodic activity [1,97]. However, different studies would have to be carried out to evaluate the most suitable methods for preparing herbal medicines for the control of diarrhoea in a bid to maximise medicinal potentials.

### 5.4. Dosages and Quality Control of the Medicines

Dosages should take into consideration the weight of the patient, the seriousness of the illness, nature of the plant, speed of absorption of medicine and the particular circumstances relative to the patient. For example, certain medicines may be highly unadvisable for pregnant women or patients with high blood pressure [98]. Most practitioners in rural communities are not aware of these facts and so need to be educated through workshops and seminars. There is usually little data made available to them on the composition and quality of most herbal medicines not only due to lack of adequate policies or government requirements but also due to a lack of adequate or accepted research methodology for evaluating traditional medicines [99]. Quality control of the finished product is very important to affirm the efficacy and purity of the medicine. At times, herbal extracts may be contaminated, adulterated, and may contain toxic compounds and so quality control of the medicines has a direct impact on their safety and efficacy [100]. 

Toxicological evaluation of medicinal plants is always neglected since prolonged and apparently uneventful use is usually considered as a testimony of safety [101]. This may not always be the case because some of the adverse effects of medicinal plant usage, such as those arising from mutagenecity may take decades to appear. It is therefore necessary to evaluate also the mutagenic effects of herbal medicines used in the treatment of diarrhoea. It would be important to study the composition and activity profiles of herbal preparations used in the management of diarrhoea so as to be able to develop appropriate management protocols in case of toxicity [102]. 

### 5.5. Packaging and Storage

Properly packaged and stored products will reduce the risk of contamination, minimize losses due to spoilage by bacteria, moulds or rodents, maintain quality and improve on the shelf life of the medicine [103]. Most herbal products deteriorate quickly when exposed to heat, light and moisture with the likelihood of fungal and bacterial growth [103]. Since herbal medicines always contain more than one active ingredient, care must be taken to determine the most suitable storage conditions of most of the components. Labelling the plants and using containers which protect the plant from contamination are both important, but are not always practiced. It is important that herbal drugs be labelled and that adequate information be carried on the label (or the package insert) to inform the users of the composition of the product, indications or actions, directions for use, cautions and adverse reactions if any, and the expiry date [104]. 

Storage can influence the physical appearance and chemical quality of plant materials and hence it is necessary to maintain appropriate storage conditions [100]. Herbal preparations could be stored in bottles (brown-coloured preferably), cans, plastic containers or sachets and locked in a dark cupboard to minimise deterioration of important phytochemical principles due to light, a prominent factor that affects phyto-formulations by generation of free radicals [96]. The container in which the medicine is stored usually depends on the nature of the plant material. Most liquid preparations are stored in bottles while powders are stored in sachets and plastic containers. The storage environment should be dry, well-ventilated, and spacious, less fungi and insects may invade rampantly. The humidity of the storehouse should be as low as possible [96]. Materials rich in volatile oils should be kept in airtight containers. Many researchers have established that the potency of powdered plant material decreases with time, with significant decreases after six months of storage [96,105,106]. For this reason, the plant material should only be powdered when needed and stability studies carried out at specific time intervals to ascertain quality [107]. 

## 6. Conclusions

It is critically important for anti-diarrhoeal agents to be readily available and attainable in rural communities of the developing world, in order to address the high morbidity and mortality among children, and for families to be educated on standard methods of application. Indigenous herbal medicines used in the treatment of diarrhoea in African rural communities are unlikely to be replaced soon by modern medicines. However, scarcity of some plant species has resulted in the use of herbal drugs with suboptimal anti-diarrhoeic activity. There is a need for sustainability of endangered species, standardization of methods of preparation and dosage, quality control of medicinal products and scientific validation of medicinal plants used in the treatment of diarrhoea in these communities. Traditional medicines have been a great source of health since pre-colonial times, and form the basis of many pharmaceutical products in the developed world. Therefore, it becomes imperative for local governments to incorporate these into the national health policy, and to make sure that certain standards are met and harmonised including the training and re-training of indigenous health practitioners. This would facilitate their professionalism, improve collaboration with other health care workers, instil confidence amongst its users who make up 80% of the population, improve quality and render the products more acceptable nationally and internationally. It could also be a good attempt to accommodate affordable local medicines and technologies in the prevention and/or treatment of diarrhoea and other diseases in Africa. 
